# Small-fragment, high turnover: soil microenvironment fluctuation effect on tree diversity in a Neotropical montane oak forest

**DOI:** 10.7717/peerj.15415

**Published:** 2023-05-23

**Authors:** Luis F. López-Calvillo, Pilar Carbó-Ramírez, Ernesto C. Rodríguez-Ramírez

**Affiliations:** 1 Laboratorio de Biogeografía y Sistemática, Facultad de Ciencias, Universidad Nacional Autónoma de México, Mexico City, CDMX, Mexico; 2 Indepent Research, Unaffiliated, Oulu, Finland; 3 Laboratorio de Dendrocronología, Continental University, Huancayo, Junín, Perú

**Keywords:** β-diversity, Cloud forest, Floristic structure, Microhabitat, Importance Value Index (IVI), Relict tree species

## Abstract

**Background:**

Soil microenvironmental variables showed an important key in *α* and β-tree diversity in Neotropical montane oak forest. Thus, understanding the microenvironment fluctuation at small-fragment effects on tree diversity is crucial in maintaining the montane oak ecosystems. In this study, we hypothesized that within a relatively small-fragment (151.63 ha), tree *α* and β-diversity fluctuate and specific soil microenvironmental factors could influence tree species diversity to answer three questions: Do tree *α* and β-diversity differ among transects, even in a short-distance between them? Do microenvironmental variables influence tree diversity composition that occurs within a relict Neotropical montane oak forest? Is there a particular microenvironmental variable influencing tree species-specific?

**Methods:**

We established four permanent transects during a year in a relict Neotropical montane oak forest, we assessed tree diversity and specific microenvironmental variables (soil moisture, soil temperature, pH, depth litterfall and light incidence). This allowed us to evaluate how microenvironmental variables at small-fragment influence *α* and β-tree diversity and tree species-specific.

**Results:**

Our results showed that *α*-diversity was not different among transects; however, β-diversity of tree species was mostly explained by turnover and soil moisture, soil temperature, and light incidence were the microenvironmental variables that triggered the replacement (*i.e.*, one species by another). Those variables also had effect on tree species-specific: Mexican beech (*Fagus mexicana*), Quebracho (*Quercus delgadoana*), Pezma (*Cyathea fulva*), Aguacatillo (*Beilschmiedia mexicana*), Pezma (*Dicksonia sellowiana* var. *arachneosa*), and Mountain magnolia (*Magnolia schiedeana*).

**Discussion:**

Our results confirm our hypothesis related to β-diversity but not with *α*-diversity; however, the tree community structure of the diversity was similar among transects. Our study represents the first effort to evaluate and link the soil microenvironmental effect on tree *α* and β-diversity, finding a high replacement in a small-fragment of Neotropical montane oak forest from eastern Mexico.

## Introduction

Neotropical montane oak forests (NMoFs; Kappelle, Cleef & Chaverri, 1992) cover ∼75% of tropical tree species and 18–25% temperate plant genera worldwide. These forests are characterized by steep ravines (>45°), topographically complex regions (from 1,000 to 2,500 m.a.s.l.; Rahbek et al., 2019; Ampoorter et al., 2020), and the presence of specific floristic dominant relict-tree composition (*Fagus mexicana* Martínez, *Magnolia schiedeana* Schltdl., *Meliosma alba* (Schltdl.) Walp*.*,*Tilia mexicana* Schltdl., and several oaks), as well as specific microenvironmental variables (*i.e.,* high pH, high moisture, and low soil temperature) play a key role in the mid-canopy structure (Rodríguez-Ramírez et al., 2021). Several studies have reported those variables as an essential driver in determining future plant diversity and health of forests (Tymen et al., 2017; Denney et al., 2020; Zou et al., 2021). For example, the amount of litter falls or canopy cover, moisture, soil pH and soil temperature are linked as components that influence the floristic structure, composition and diversity in forest ecosystems (Barik et al., 1992; Francisco et al., 2021).

Local microenvironmental variables influence small-scale (<1 m) on tree *α* (richness and ecological diversity) and β-diversity (turnover and nestedness) heterogeneity and plant distribution patterns (Rodríguez-Ramírez, Sánchez-González & Ángeles Pérez, 2018; Morandi et al., 2020). Specifically, turnover is a relevant component of β-diversity that influences the tree species with restricted distribution, reflecting particular processes as an adaptation to microenvironmental variation (Swenson, Anglada-Cordero & Barone, 2011; Denney et al., 2020).

The Mexican NMoFs is characterized by isolate and archipelagic distribution, as well as high tree diversity including ∼164 relict-endemic species (Valencia, 2017; Carrero et al., 2020); nevertheless, NMoF microenvironmental variation is poorly understood, and can influence tree-dominant species richness and diversity (Anderson et al., 2011; Fahey, Sherman & Tanner, 2016). In this study, we hypothesized that within a relatively small-fragment (151.63 ha), tree *α* and β-diversity fluctuate and specific soil microenvironmental factors could influence tree species diversity to answer three questions: (1) Do tree *α* and β-diversity differ among transects, even in a short-distance between them? (2) Do microenvironmental variables influence tree diversity composition that occurs within a relict Neotropical montane oak forest? (3) Is there a particular microenvironmental variable influencing tree species-specific?

## Materials & Methods

### Sampling site

**Figure 1:**
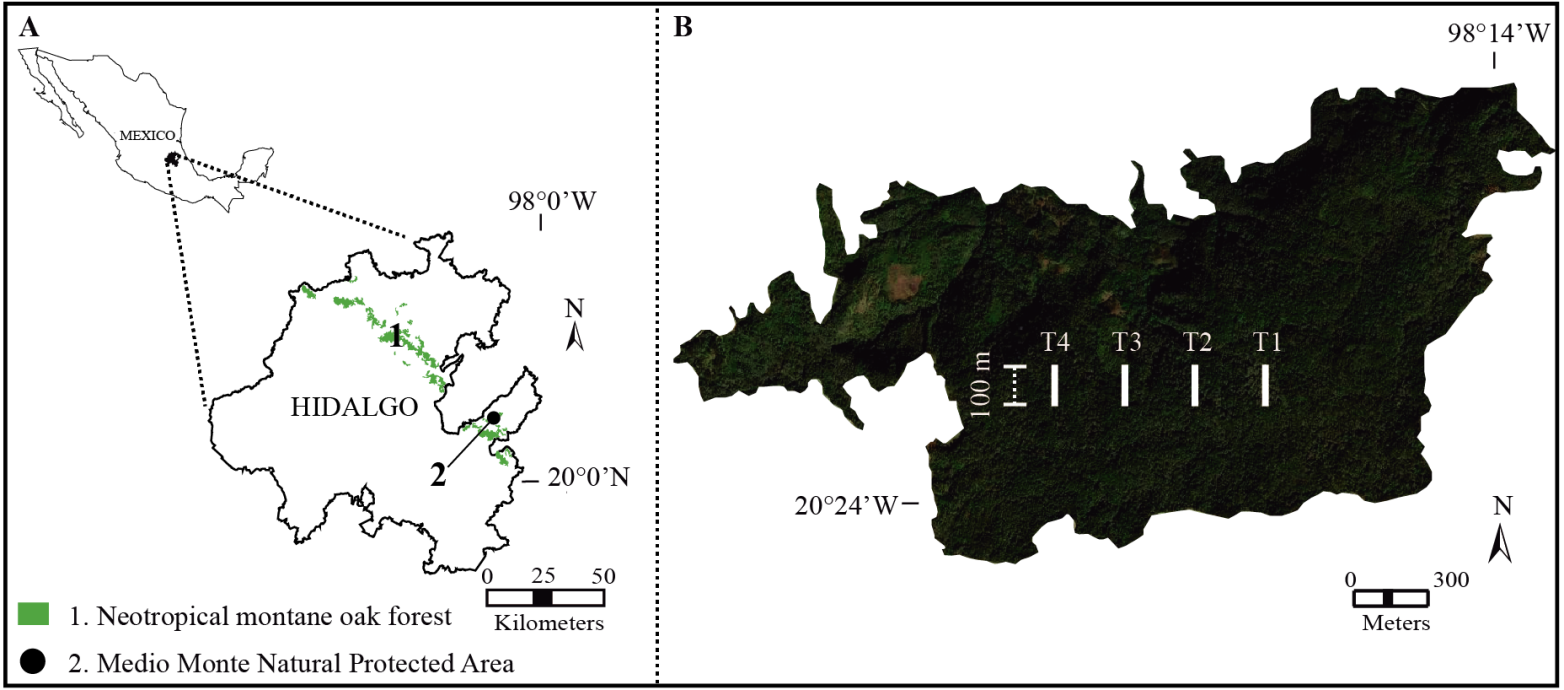
Study area. (A) Distribution of Neotropical montane oak forest in Hidalgo state, Mexico. (B) Geographical location of the study transects (white lines) in Medio Monte Natural Protected Area.

The study was performed in the Medio Monte Natural Protected Area (San Bartolo Tutotepec, Hidalgo state, Mexico; 20°24′51.50″N, 98°15′32.07″W; 1,800–1,944 m.a.s.l.; Fig. 1A). The study forest is the largest continuous relict-NMoF in eastern Mexico (ca. 151.63 ha; SEMARNAT & Secretaría del Medio Ambiente y Recursos Naturales, 2010) with rugged terrain with steep slopes >40°. The climate is temperate (Cwb *sensu* Peel, Finlayson & Mcmahon (2007)) with three distinct seasons throughout the year: dry cool October-January, dry warm early February-May, and humid June-September. Average annual temperature 12.7−14 °C (Rodríguez-Ramírez et al., 2021), the total annual precipitation is 1,200–2,015 mm, and relative moisture is 60–85% (Miranda & Sharp, 1950). The buried soil is covered by younger deposits (Andosol-Humic; IUSS Working Group, 2022). The study forest displays pH values of 4–6 (Peters, 1995).

### Study transects and tree inventory

During 2019 from January to December, we established and geo-referenced (with a GPS; Garmin eTrex^®^ 10; Garmin Ltd., Olathe, KS, USA) four permanent transects (as far as possible from anthropic activities and roads) with a length of 100 m and ∼300 m apart in the study forest (Fig. 1B). To identify the structural importance of tree species, at each transect five sample plots (20 × 20 m) were set up and 20 m apart to assure data independence (Roberts-Pichette & Gillespie, 1999). Conspicuous tree species with a diameter at breast height (DBH) ≥ 1.5 cm were collected, identified and quantified every three months (Rodríguez-Ramírez, Sánchez-González & Ángeles Pérez, 2018). Tree species were identified using specific taxonomic keys (Rzedowski, 2015) and the vascular plant species nomenclature was updated at the Tropicos website (Missouri Botanical Garden; http://www.tropicos.org). The specimens were deposited in the FES–Iztacala–UNAM herbarium, Mexico (IZTA; https://www.zaragoza.unam.mx/herbario/).

### Microenvironmental variables

We measured five microenvironmental variables tri-monthly in each plot between 8:00 and 17:00 to normalize the daily fluctuation (Table 1): soil moisture, soil temperature, soil pH, depth litterfall, and light incidence (see Rodríguez-Ramírez, Sánchez-González & Ángeles Pérez, 2018 for more details). Four measurements were taken at the four cardinal points (*e.g.*, north, south, east, and west) of each plot. The values were averaged for statistical analysis.

### Analyses vegetation

We assessed the Importance Value Index (IVI; express the dominance and biological success of specific species; Susilowati et al., 2020) of each tree species and performed rank-abundance curves to identify the structure roll of each tree species among transects, estimated by summing relative frequency, density and dominance (Naidu & Kumar, 2016):

IVI = ($ \frac{1}{2} $) relative BA + relative D.

We calculated basal area (BA) using the formula BA = *π*r^2^, where *π* = 3.1416 and r = (0.5) (DBH) is the radius of the tree; and relative density (D) was determined using the number of individuals of each species (Mostacedo & Fredericksen, 2000). To characterize the tree structure and composition of each transect, we developed semi-realistic Richards profile diagrams using the software Adobe Illustrator *v.* 23.0.5 (http://www.adobe.com).

### Diversity analyses

To ensure that our tree survey effort was enough to record a significant sample for the inventory, we used sample coverage estimates suggested by Chao & Jost (2012), ranging from 0 (minimum) to 100% (maximum). Tree diversity was estimated using *q* = 0 (species richness) and *q* = 1 (species diversity) (Hill, 1973). We performed this estimation among transects. The *α*-diversity, for a given diversity order *q*, is given by:

\begin{eqnarray*}{q}_{D}= \left( \begin{array}{@{}ll@{}} \displaystyle \sum _{i=1}^{s}&\displaystyle {p}_{i}^{q} \end{array} \right) ~1/(1-q) \end{eqnarray*}

**Table 1:** Overview of soil microenvironment variables and their measurement method (see Rodríguez-Ramírez, Sánchez-González & Ángeles Pérez, 2018 for more details).

**Microenvironmental variables**	**Measurement**
Soil moisture (%) in volume	Recorded at a depth of 2–3 cm, with a hygrometer (VT–05) that includes a scale from 1 to 8 (10% to 80% moisture).
Soil temperature (°C)	Measured with a soil thermometer (Taylor^®^ Switchable Digital Pocket Thermometer) at a depth 5 cm.
Depth litter fall (cm)	Measured using a scaled metal ruler. It was drilled down to the humus of the soil layer.
Soil pH (0-14)	Recorded with a pH soil meter (ANGGREK^®^) at a depth 5 cm, with an accuracy of ± 0.2 pH.
Light incidence (%)	Estimated using a concave mirror densitometer (Forestry Suppliers Spherical Crown Densitometers, Model A).

where *S* is the number of species, *pi* is the comparative abundance of species *i*, and *q* is the order number of diversity. We performed rarefaction curves to standardize samples that differ in terms of individual size or plot size (Chao, KH & Hsieh, 2016). They were compared with 95% confidence intervals. We performed the analysis using the iNEXT R-library (Chao et al., 2014; https://chao.shinyapps.io/iNEXTOnline/).

We partitioned β-diversity according to the procedure of Podani & Schmera (2011), which is based on the approach of Baselga (2010). According to this method, total dissimilarity (βcc) is 1-minus Jaccard similarity coefficient. βcc is divided into two components: turnover (β.3, replacement of one species by another or loss of species) and nestedness (βrich, differences in species richness). The β-diversity was assessed using the IVI of each tree species (Naidu & Kumar, 2016). These analyses were performed in R-software *v.* 2.4.1 (R Core Team, 2018), using the R-script of Carvalho, Cardoso & Gomes (2012).

### Linking microenvironmental variables with turnover tree diversity

We performed a principal component analysis (PCA) using tree abundance data to identify the most strongly discriminating soil microenvironmental variables (Anderson et al., 2011; Kent, 2011). All microenvironmental variables were first transformed to log10 after adding a constant because each variable showed different value types (Kindt & Coe, 2005). We performed a redundancy analysis (RDA; Borcard, Gillet & Legendre, 2011) to analyze the relationship between turnover tree diversity (IVI data) and microenvironmental variables among four transects. We intend the technique to reveal major trends in the variation of a multidimensional dataset in a reduced space of selected, linearly independent dimensions (Legendre & Legendre, 1998). We achieved the analyses with the R-software and *vegan* package (Oksanen et al., 2016).

Based on the IVI tree species rank-abundance curves results, we performed a quasi-Poisson generalized linear model (GLM; Ver Hoef & Boveng, 2007) to determine specific microenvironmental variables that could influence on turnover of tree species with high IVI values; the tree species as dependent variables, and microenvironmental variables as independent factors. Specific predictor microenvironmental variables were log or square root transformed to address non-normality (Shapiro-Wilk test; Hanusz, Tarasinska & Zielinski, 2014). In all cases, we selected the best model fitted for each microenvironmental variable; we used the Adjusted Akaike’s information criteria (AICc) selecting the best model with the minimum AICc value (Hurvich & Tsai, 1989). Statistically significant variables are *P* < 0.05 and *P* < 0.01 (Borcard, Gillet & Legendre, 2011). We achieved all GLM analyses through using R-software with the *glm2*-function in *stats* package. Besides, response curves of six tree species with high IVI values against specific soil microenvironmental variables were performed with CANOCO *v.* 5.0 (Šmilauer & Lepš, 2014).

## Results

### Floristic composition

**Figure 2:**
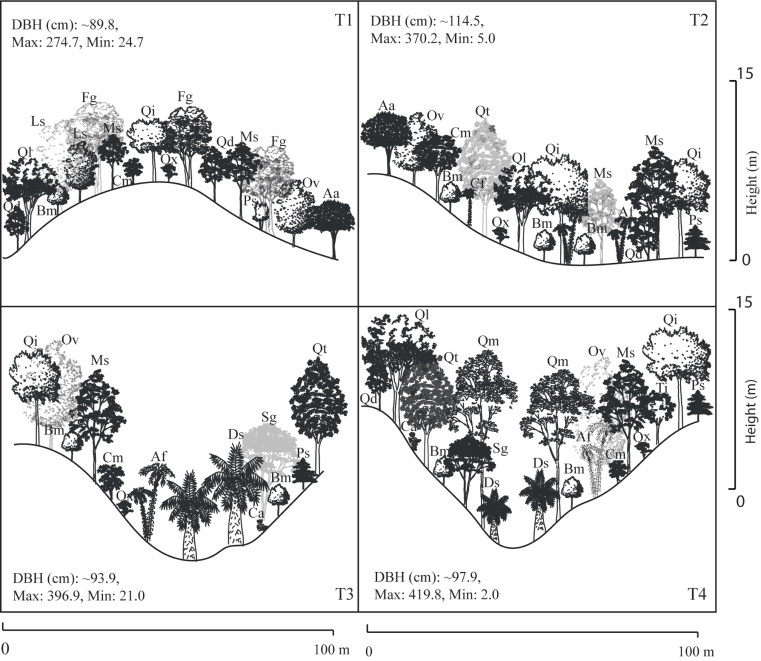
Stand profile diagrams. Profile diagram illustration changes in tree species composition and structure among Neotropical montane oak forest transects (T1, T2, T3 and T4).

**Figure 3:**
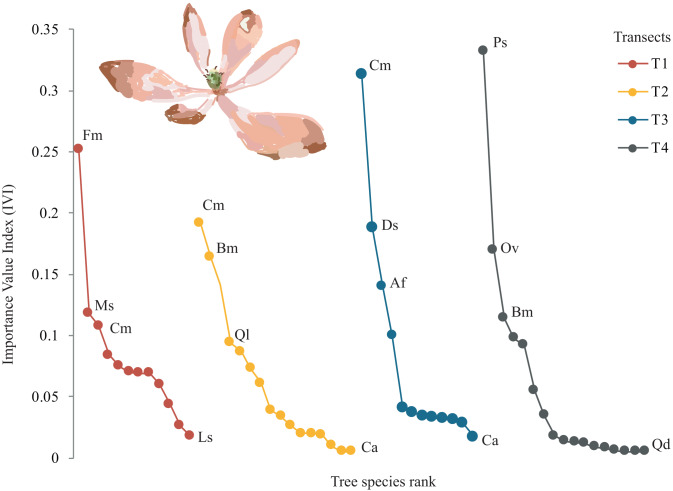
Rank curves showing tree species classified for Importance Value Index (IVI) in each transect.

A total of 22 tree species were identified in the four transects (Table 2). The vegetation profiles allowed to detect dissimilarity floristic species structure and composition among transects (Fig. 2). The average tree diameter (DBH) in the transect T1 was 89.8 cm; T2 = 114.5 cm; T3 = 93.9 cm; and T4 = 97.9 cm (Fig. 2). Overall, the Importance Value Index (IVI) showed ranges from 0.333 (*Prunus serotina* Ehrh.) to 0.005 (*Conostegia arborea* Steud.) (Table 2, Fig. 3). By each transect the rank-curve showed different vegetation composition and structure dominated by: T1, Mexican beech (IVI = 0.252); for T2 and T3, Mexican *Clethra* (IVI = 0.192, 0.313 respectively); and T4, Wild black cherry (IVI = 0.333), (Fig. 3). The uncommon tree species were: for T1, Sweetgum (*Liquidambar styraciflua* L.; IVI = 0.018); for T2 and T4, Capulín (IVI = 0.005); and T3, Quebracho (*Quercus insignis* M. Martens & Galeotti; IVI = 0.017), (Fig. 3).

**Table 2:** Importance Value Index (IVI) of tree species among Neotropical montane oak forest transects. Bold numbers represent the highest IVI values in each transect.

**Code**	**Tree species**	**Neotropical montane oak forest transects**			
		**T1**	**T2**	**T3**	**T4**
Aa	*Alnus acuminata* Kunth	0.069	0.006	0	0
Af	*Alsophila firma* (Baker) D.S. Conant	0	0.039	0.140	0.093
Bm	*Beilschmiedia mexicana* (Mez) Kosterm.	0.026	0.164	0.032	0.114
Cm	*Clethra mexicana* DC.	0.108	**0.192**	**0.313**	0.036
Ca	*Conostegia arborea* Steud.	0	0.005	0.042	0.005
Cf	*Cyathea fulva* (M. Martens & Galeotti) Fée	0	0.034	0	0
Ds	*Dicksonia sellowiana* var*. arachneosa* Sodiro	0	0	0.188	0.009
Fm	*Fagus mexicana* Martínez	**0.252**	0	0	0
Ls	*Liquidambar styraciflua* L.	0.018	0	0	0
Ms	*Magnolia schiedeana* Schltdl.	0.118	0.074	0	0.099
Ox	*Oreopanax xalapensis* (Kunth) Decne. & Planch.	0.084	0.011	0.037	0.012
Ov	*Ostrya virginiana* (Mill.) K. Koch	0.070	0.021	0.028	0.170
Po	*Perrottetia ovata* Hemsl.	0	0	0	0.014
Pa	*Persea americana* Mill.	0	0.142	0	0
Ps	*Prunus serotina* Ehrh.	0.075	0.061	0.033	**0.333**
Qd	*Quercus delgadoana* S. Valencia, Nixon & L.M. Kelly	0.060	0.019	0	0.056
Qi	*Quercus insignis* M. Martens & Galeotti	0	0.020	0.017	0.006
Ql	*Quercus laurina* Bonpl.	0	0.088	0	0
Qm	*Quercus meavei* S. Valencia, Sabas & O.J. Soto	0	0	0	0.009
Qt	*Quercus trinitatis* Trel.	0	0	0	0.007
Sg	*Styrax glabrescens* Benth.	0	0	0.034	0.018
Ti	*Turpinia insignis* (Kunth) Tul.	0	0	0	0.013

### Tree diversity

**Figure 4:**
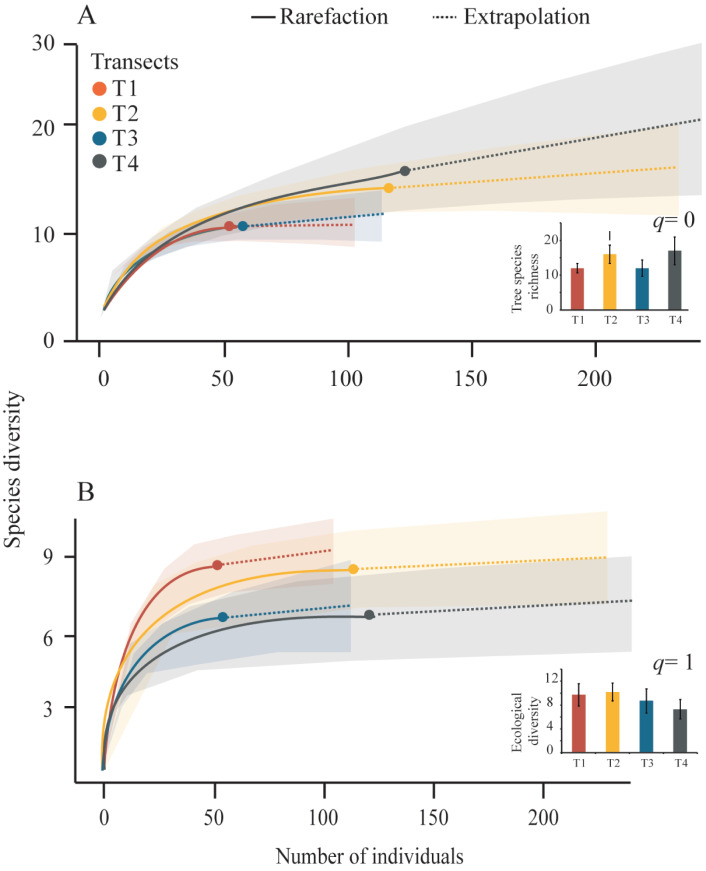
Rarefaction curves and Hill-number graphs. Rarefaction curves for the sampled Neotropical montane oak forest transects. The inner graphs show the Hill-number with 95% confidence intervals. Tree species richness (*q* = 0) and species diversity (*q* = 1) were calculated accounting for each transect. The error bars represent the standard deviation.

We obtained tree diversity completeness >90% in each transect (Table 3). The tree *α*-diversity values (*q* = 0, tree species richness; and *q* = 1, ecological diversity) and rarefaction curves were not significantly different among transects (Table 3, Fig. 4). The T4 showed the highest richness (17 species) and the lowest richness values were registered in T1 and T3 (12 species), (Fig. 4A). The highest tree ecological diversity was found in T2 (10.18) and the lowest value was detected in T4 (7.3), (Fig. 4B).

### β-diversity

**Figure 5:**
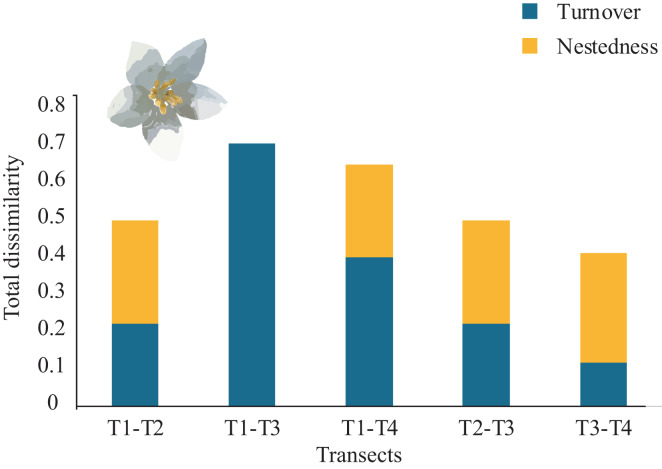
β-tree species composition (turnover and nestedness). Total dissimilarity in β-tree species composition (turnover and nestedness) between pairs of four Neotropical montane oak forest transects.

Overall, the total average tree β-diversity (βcc) among transects was ∼0.55; turnover (β.3) ∼0.36 and nestedness (βrich) ∼0.18. Considering all pairs of comparisons of all sampled transects, partitioning total β-diversity, we found that between T1-T3 and T2-T4 were explained by turnover (β.3 = 0.70 and 0.54 respectively) and null nestedness value for both pairs (βrich = 0.00). Similarly, the relationship between T1-T4 was explained by turnover (β.3 = 0.40) and low nestedness (βrich = 0.25). The pairs that showed similar values of turnover and nestedness were between T1-T2 and T2-T3 (β.3 = 0.22; βrich = 0.27). The lowest turnover and nestedness values were between T3-T4 (β.3 = 0.11; βrich = 0.29), (Fig. 5). Each transect showed unique tree species composition such as Mexican beech (*Fagus mexicana*, T1), Sweetgum (*Liquidambar styraciflua*, T1), Pezma (*Cyathea fulva* (M. Martens & Galeotti) Fée, T2), Palo de agua (*Perrottetia ovata* Hemsl., T4), Wild avocado (*Persea americana*, T2), Palo verde (*Turpinia insignis* (Kunth) Tul., T4) and Querbrachos (*Quercus delgadoana*, T2; *Q*. *meavei*, T4; and *Q*, *trinitatis*, T4).

**Table 3:** Hill’s numbers (*q* = 0, *q* = 1), Confidence Interval (CI), and sample coverage values (Sc) of tree species among four Neotropical montane oak forest transects.

	*q* = 0	CI	*q* = 1	CI	Sc
**Transects**					
T1	12	1.34	9.71	1.89	0.99
T2	16	2.64	10.18	1.5	0.97
T3	12	2.34	8.67	02.03	0.97
T4	17	3.96	7.3	1.63	0.93

### Local microenvironmental variables effect on tree diversity

**Figure 6:**
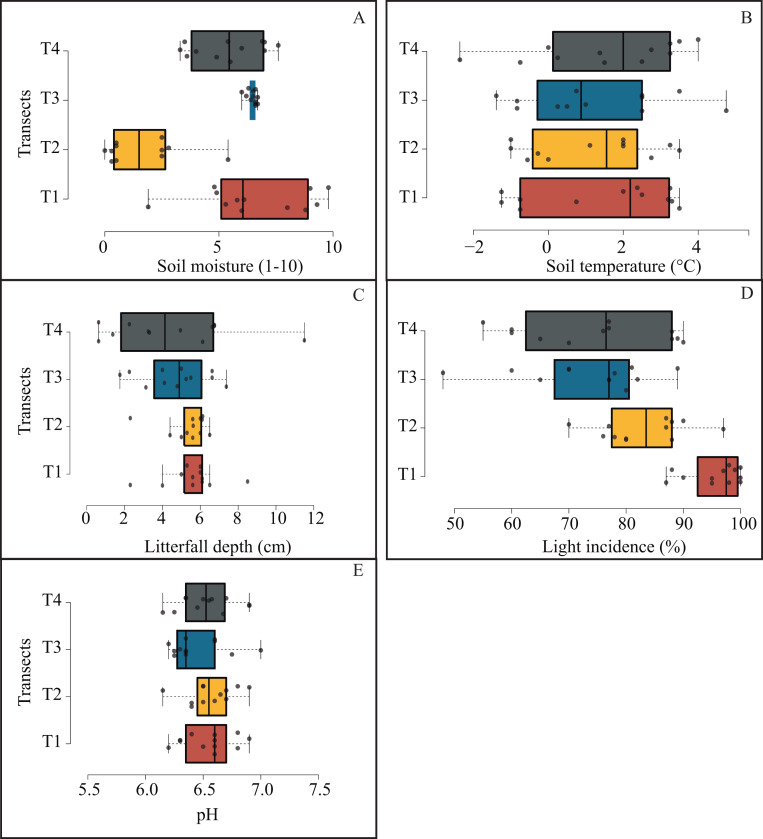
Box plots jittered showing the microenvironmental variation among transects. Solid line represents the average and numbers within each box indicate statistically significant differences among transects.

Microenvironmental values were not differing significantly among transects (Fig. 6); notwithstanding, we got the most contrasting light incidence values where T1 recorded ∼98% and T4 ∼77% (Fig. 6D); and we recorded the opposite tendency in pH values among transects (from 6.3 to 7; Fig. 6E).

**Figure 7:**
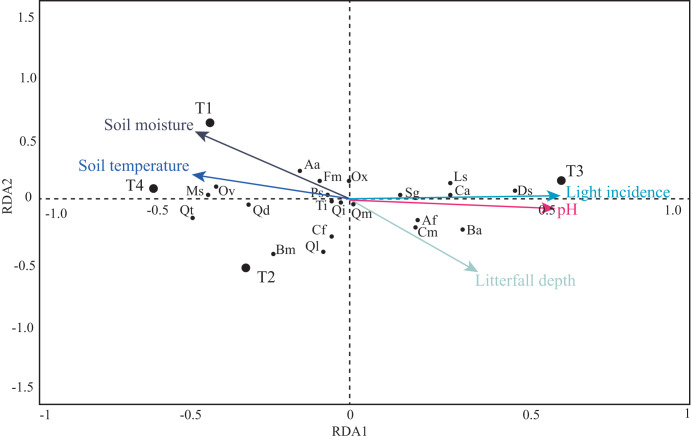
Projection of soil microenvironmental variables, transects and tree species in the RDA. The length and direction of the arrows show the relative degree and direction of association. Tree species codes are in Table 2.

**Figure 8:**
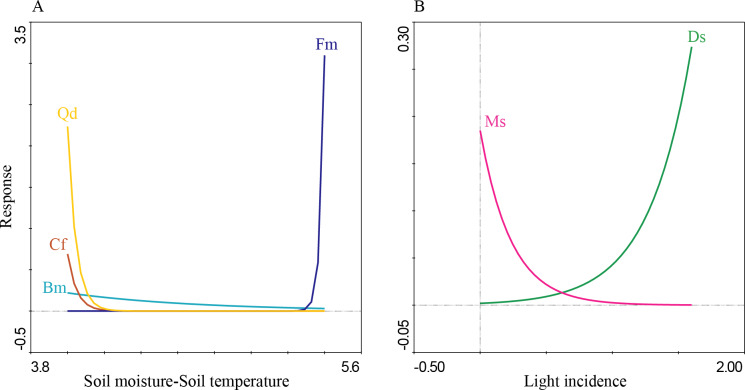
Response curves of six tree species with high IVI values against specific soil microenvironmental variables in the study forest. (A) Soil moisture-soil temperature; and (B) light incidence.

The first four PCA axes explained 80% of the microenvironmental variation. Moreover, the global permutation test of redundancy analysis ordination (RDA) displayed that the relationship between tree species and microenvironmental variables (*i.e.,* soil moisture, soil temperature, litterfall depth, light incidence, and pH) were significant (*P* < 0.01). The first two axes explained 95% of the variance in tree species and between tree species and soil microenvironmental relationship (Table 4; Fig. 7). Soil moisture influences tree composition in the T1, soil temperature on tree composition in the T4, and light incidence on tree composition in the T3 (Fig. 7). Quasi-Poisson generalized linear model analysis (GLM) showed that soil microenvironmental variables significantly influenced specific tree species; Mexican beech (*Fagus mexicana*), white Quebracho (*Quercus delgadoana*), Pezma (*Cyathea fulva*) and Aguacatillo (*Beilschmiedia mexicana*) were influenced by soil moisture and soil temperature; while Mountain magnolia (*Magnolia schiedeana*) and Pezma (*Dicksonia sellowiana* var. *arachneosa* (Sodiro)) were influenced by the light incidence (Table 4; Fig. 8).

## Discussion

Our results confirm our hypothesis related to β-diversity but not with *α*-diversity; nevertheless, tree community structure of the diversity was similar among transects in terms of *q* = 0 and *q* = 1 values. Likewise, Monge-González et al. (2020), Morandi et al. (2020), and Monarrez-González et al. (2020) found that *α*-diversity was not fluctuating in Neotropical montane forests, possibly linked with particular microenvironmental variables as temperature and moisture fluctuations through transects. Meanwhile, β-diversity results were explained by turnover (β.3) overall and by pairs (T1-T3, T1-T4 and T2-T4) in a small-fragment; previous studies have related turnover (β.3) to micro-habitat heterogeneity (Baldeck et al., 2016; Tymen et al., 2017; García-Hernández et al., 2019). Tree turnover may reflect particular ecological processes, such as restricted species dispersion (large and small-scales), adaptation to specific microenvironmental variation, delaying response to climate and anthropic effect (Condit et al., 2002).

Even though nestedness (βrich) were not significantly different among transects because any pair comparison showed high values (Fig. 5), the βrich showed a non-random process of species loss resulting as subsets of tree species at higher-richness transect (Baselga, 2010) and in nature is driven by isolation, limiting dispersal, and availability of suitable habitat at a small-scale influencing extinction-establishment dynamics by microenvironmental tolerance (McAbendroth et al., 2005; Monge-González et al., 2020). We found that even a small-fragment *α* and β-diversity showed robustness given the completeness of our inventory (>90%).

The results related with the soil microenvironmental variables, overall; we found that soil moisture, light incidence and soil temperature are an important driver on tree turnover; these variables were reported as highly sensitive to abrupt floristic structure turnover on specific forest communities (Williams-Linera et al., 1996; Fahey, Sherman & Tanner, 2016). According to the IVI values and tree species life-traits and composition we identified a pool of sensitive dominant tree species strongly influenced by soil moisture, soil temperature and light incidence: Aguacatillo (*Beilschmiedia mexicana*), Mexican beech (*Fagus mexicana*), Mountain magnolia (*Magnolia schiedeana*), Pezmas (*Cyathea fulva* and *Dicksonia sellowiana* var. *arachneosa*) and Quebracho (*Quercus delgadoana*). These results are congruent with those reported by Rodríguez-Ramírez, Sánchez-González & Ángeles Pérez (2018), where Mexican beech and other Tropical Montane Cloud Forest Tree species were associated with soil moisture, soil temperature, the quantity of litter, litter depth, soil pH and canopy cover. Another study, Hammond et al. (2020) found that soil temperature influenced the regeneration on European beech (*Fagus sylvatica* L.) and the moisture on Norway spruce (*Picea abies* (L.) H. Karst.). Otherwise, the tree species mentioned above have been reported as characteristics of preserved low disturbance environments (Williams-Linera et al., 1996); hence, we could conclude that transect T1 was the site best preserved. On the other hand, the transects T2, T3 and T4 were anthropic or naturally influenced because of the presence of Mexican Clethra (*Clethra mexicana*) and Wild black cherry (*Prunus serotina*). Mexican Clethra is a tree species much broader which makes it tolerant to the microenvironmental variations (*e.g.*, high light incidence, low soil moisture, and high soil temperature; Monterroso-Rivas, Gómez-Díaz & Tinoco-Rueda, 2012). Likewise, Wild black berry is a key tree in oak forest dynamics because of accelerated growth and sprouting capacity (Auclair & Cottam, 1971; Esch & Kobe, 2021). These tree species are characteristics of secondary forest, influenced by light incidence (T3) and soil temperature (T4). Monge-González et al. (2020) found similar results, where the light incidence and soil temperature promoting fast-growing over shade-tolerant and slow-growing species, which are well adapted to secondary forests; while Fisher et al. (2013) define these variables as essential for several soil processes and reactions that may include water and nutrient uptake, microbial activities, nutrient cycling, root growth and many other processes.

**Table 4:** Summary of quasi–Poisson generalized linear model (GLM), with soil microenvironmental variables effect on high IVI values of tree species. Bold values represent significant differences.

	**Tree species with high IVI values**												
	*Fagusmexicana* (Fm)		*Quercus laurina* (Ql)		*Magnolia schiedeana* (Ms)			*Dicksonia sellowiana* var. *arachneosa* (Ds)		*Cyathea fulva* (Cf)		*Beilschmiedia mexicana* (Bm)	
**Microenvironmental variables**	*X* ^2^	*P*	*X* ^2^	*P*	*X* ^2^	*P*	*X* ^2^		*P*	*X* ^2^	*P*	*X* ^2^	*P*
Soil moisture	2.266	0.028^*^	−1.943	**0.058** **⋅**	1.573	0.123	−0.44				0.662		−1.943	**0.058** **⋅**	−1.87	**0.068** **⋅**
Soil temperature	1.75	**0.087⋅**	−2.148	0.037^*^	0.717	0.477	0.063		0.95	−2.148	0.037^*^	−2.04	0.047^*^
Litterfall depth	−0.376	0.709	0.694	0.491	−0.503	0.617	−0.319				0.751		0.694	0.491	0.505	0.616
Light incidence	−1.092	0.281	−0.488	0.628	−1.875	**0.067** **⋅**	1.695		**0.097** **⋅**	−0.488	0.628	−0.971	0.337
pH	1.135	0.263	−0.116	0.908	1.37	0.178	−1.17				0.248		−0.116	0.908	0.008	0.993

**Notes.**

* *P* < 0.05, <0.01· values.

Even, our results are limited to one NMoF small-fragment, according to our diversity results, IVI values, and microenvironment variables, we could suggest that not only the species richness provide enough information to decide about protecting relevant microrefugia as well as assess the quality and stability of the habitat when used small-scale microenvironmental data. For example, *α*-diversity showed no difference among transects, but T4 had the highest richness; while β-diversity components showed high turnover values between T1-T3, T1-T4 and T2-T4 where real species replacement occurred. Therefore, we cannot consider only as an important microrefugia the T4, otherwise, consider the other transects too; then, with all these elements we can recommend areas with potential conservation to local authorities by endangered relict communities as in T1.

## Conclusions

This study represents the first effort to measure *α* and β-diversity, as well as linked to soil microenvironmental effects on tree species diversity in a relict Neotropical montane oak forest in eastern Mexico. We concluded that high IVI values and turnover tree diversity in a small-scale is influenced by soil moisture, soil temperature and light incidence variation. Therefore, microhabitats with specific environmental features in the NMoF could influence the establishment of relict-tree species; however, the abiotic stresses imposed by rapid anthropogenic influence and climate change in the long-term, could affect the ecosystem stability. According to Kappelle (2006), the NMoF is an important ecosystem, recognized as a multifunctional forest (*e.g.*, timber source, water production, carbon sink and reservoir, and landscape of great scenic beauty) and contains a highly endangered and endemic species, so it is important to find a healthy balance between use and conservation. We need additional research effort to understand the dynamic at regional-scale in the Mexican Neotropical montane oak forests.

##  Supplemental Information

10.7717/peerj.15415/supp-1 Supplemental Information 1 Raw data exported from the fieldwork applied for data alpha and beta analyses and preparation for Figs. 2–8 and Tables 2–4

10.7717/peerj.15415/supp-2 Supplemental Information 2 Raw data exported from the fieldwork applied for data alpha and beta analyses and preparation for Figs. 6–8 and Table 4

10.7717/peerj.15415/supp-3 Supplemental Information 3 Raw data exported from the field work applied for data RDA analyses and preparation for Figs. 7–8 and Table 4

## Additional Information and Declarations

### Data Availability

The following information was supplied regarding data availability: Database of the species and analyses performed.
